# 5-Aminolevulinic Acid Attenuates Glucose-Regulated Protein 78 Expression and Hepatocyte Lipoapoptosis via Heme Oxygenase-1 Induction

**DOI:** 10.3390/ijms222111405

**Published:** 2021-10-22

**Authors:** Takaaki Hashimoto, Takaaki Sugihara, Tsutomu Kanda, Tomoaki Takata, Hajime Isomoto

**Affiliations:** 1 Faculty of Medicine, School of Medicine, Tottori University, Yonago 683-8503, Japan; b17m1070u@edu.tottori-u.ac.jp; 2 Division of Medicine and Clinical Science, Department of Gastroenterology and Nephrology, Faculty of Medicine, Tottori University, Yonago 683-8504, Japan; tsutomu-k@tottori-u.ac.jp (T.K.); t-takata@tottori-u.ac.jp (T.T.); isomoto@tottori-u.ac.jp (H.I.)

**Keywords:** endoplasmic reticulum stress, heme oxygenase-1, glucose-regulated protein 78, steatohepatitis, 5-aminolevulinic acid

## Abstract

Endoplasmic reticulum (ER) stress plays a pivotal role in the progression of steatohepatitis. 5-aminolevulinic acid (5-ALA), a precursor in the heme biosynthetic pathway, has recently been reported to induce heme oxygenase (HO)-1. HO-1 exerts important cytoprotective actions. In this study, we aimed to explore the therapeutic potential of 5-ALA on palmitate-induced ER stress and lipoapoptosis. Huh-7 cells were treated with palmitic acid (PA) (800 μM) to induce steatosis for eight hours. Steatosis was evaluated by Lipi-green staining. 5-ALA (200 μM) was added with PA. The gene expression levels of the nuclear factor erythroid 2–related factor 2 (*NRF2*), *HO-1*, Glucose-regulated protein 78 (*GRP78*), activating transcription factor 6 (*ATF6*), PKR-like endoplasmic reticulum kinase (*PERK*), inositol-requiring enzyme 1 (*IRE1*), C/EBP homologous protein (*CHOP*), and B-cell lymphoma 2 (*BCL-2*) were evaluated by RT-PCR. Caspase-3/7 activity was evaluated by fluorescein active Caspase-3/7 staining. Cell death was evaluated by Annexin V/SYTOX green staining. PA significantly induced steatosis and increased *GRP78* expression in Huh-7 cells. 5-ALA significantly induced *HO-1* and decreased *GRP78* expression. *ATF6* was subsequently decreased. However, *NRF2* and *CHOP* expression were not altered. Anti-apoptotic *BCL-2* expression significantly increased, and Caspase 3/7 activity and cell death also decreased. 5-ALA has a therapeutic potential on hepatic steatosis by suppressing ER stress and lipoapoptosis by attenuating *GRP78* via *HO-1* induction.

## 1. Introduction

Nonalcoholic fatty liver disease (NAFLD) has recently emerged as a common public health problem. The prevalence of NAFLD is approximately 25% of the global population [1], which is increasing in the Asia-Pacific region [2]. The multiple parallel hits hypothesis by Tilg et al. [3], a current concept regarding NAFLD pathogenesis, indicated several contributing factors to this disease, including endoplasmic reticulum (ER) stress.

The ER is the largest organelle in the cell and is a major site of protein synthesis and transport, protein folding, lipid and steroid synthesis, carbohydrate metabolism, and Ca^2+^ storage [4]. Disruption to protein folding or Ca^2+^ homeostasis in the ER leads to the accumulation of unfolded proteins, a condition known as ER stress. The mechanism of ER stress was explored by Kazutoshi Mori and Peter Walter in the 1990s [5]. ER stress leads to activation of the unfolded protein response (UPR) pathway to maintain protein homeostasis [6]. The UPR is controlled by glucose-regulated protein 78 (GRP78) and three UPR transducers: activating transcription factor 6 (ATF6), PKR-like endoplasmic reticulum kinase (PERK), and inositol-requiring enzyme 1 (IRE1). GRP78 binds directly to the transducers under normal conditions and keeps inactive [6]. Persistent activation of the UPR, on the other hand, is associated with the pathogenesis of a number of diseases [7,8,9,10,11]. 

Some studies, including ours, have demonstrated that obesity induces ER stress and plays a pivotal role in both the development of steatosis and the progression to steatohepatitis [12,13,14]. Tilg et al. [3] also indicated that interfering with ER stress might be a future treatment option.

Our group previously conducted a study using 5-aminolevulinic acid (5-ALA) as a precursor of protoporphyrin IX (PpIX, a photosensitizer) to detect early gastric cancer by laser-based photodynamic endoscopy [15]. 5-ALA, ubiquitous in living organisms, is a precursor of tetrapyrroles such as chlorophyll, vitamin B12, and heme [16]. 5-ALA induces heme oxygenase (HO)-1 and attenuates apoptosis [17,18,19]. HO-1 is a heme degrading enzyme, which degrades heme into carbon monoxide (CO), biliverdin, and divalent iron ion (Fe2^+^) [20]. HO-1 is also an anti-inflammatory enzyme [21,22,23]. HO-1 expression is upregulated in the liver tissue of NASH patients [24]. The physiological elevation of HO-1 in NASH does not inhibit apoptosis due to lipotoxicity. However, HO-1 induction can reportedly prevent ER stress-mediated cell death [25].

5-ALA has already been approved by the European Medicines Agency (EMA) and the United States Food and Drug Administration (FDA) as an optical imaging agent for use in people affected by high-grade gliomas [26,27]. It can be administered orally and has a good safety profile [26]. It is a good candidate for drug repositioning. 

In this study, we aimed to explore the therapeutic potential of 5-ALA on palmitate-induced ER stress and lipoapoptosis in the Huh-7 cell line as a human hepatocyte model.

## 2. Results

### 2.1. Palmitate-Induced Steatosis 

**Figure 1:**
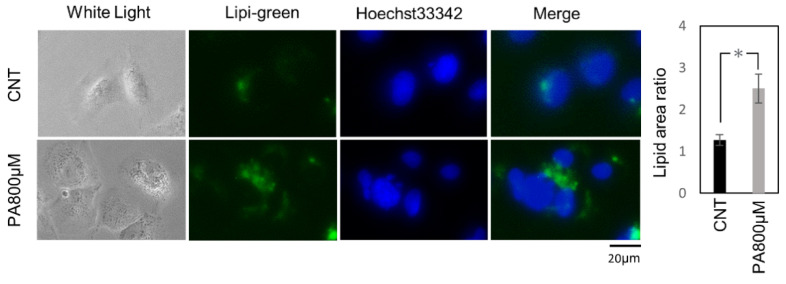
Palmitate-induced steatosis. Representative fluorescence images of Lipi-green staining on Huh-7 cells treated with 1% BSA for the controls or 800 μM of palmitic acid for the treatment group. The results were expressed as the ratio compared with the control. The quantification is based on ten randomly captured fields in each group. Bars indicate average ± SEM. * *p* < 0.05. CNT, control; PA, palmitic acid.

First, to investigate the optimal concentration of palmitic acid (PA) for inducing steatosis in the Huh-7 cell line, these cells were treated with 200, 400, 600, and 800 μM of PA. Lipi-green (LD-02, Dojindo Laboratories, Kumamoto, Japan) could be used to depict the accumulation of lipids in the cytosol. Finally, we found that 800 μM of palmitic acid for eight hours could induce significant steatosis in Huh-7 cells (Figure 1).

### 2.2. 5-ALA Attenuated GRP78 and Increased Bcl-2 Expression via Inducing HO-1 Expression

**Figure 2:**
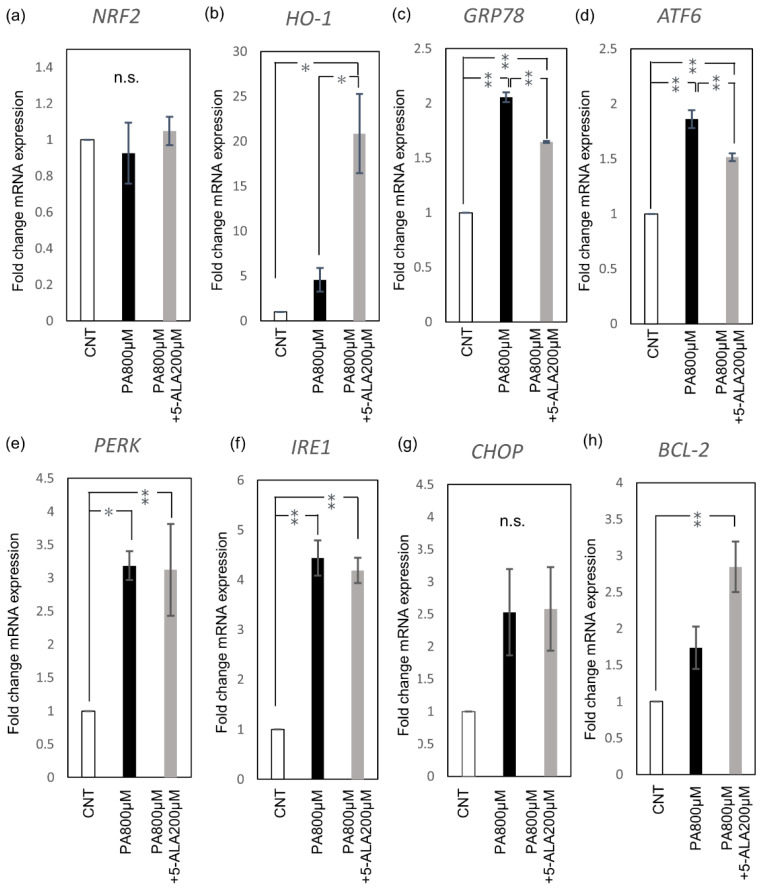
Redox and ER stress-associated gene expression. Huh-7 cells were treated with 1% BSA as the control and 800 μM of palmitic acid with or without 200 μM of 5-ALA for eight hours as the treatment groups. (**a**) *NRF2*, (**b**) *HO-1*, (**c**) *GRP78*, (**d**) *ATF6*, (**e**) *PERK*, (**f**) *IRE1*, (**g**) *CHOP*, and (**h**) *BCL-2* expression. The results were expressed as the ratio compared with the control. Bars indicate average ± SEM. * *p* < 0.05, ** *p* < 0.01. CNT, control; PA, palmitic acid; *NRF2*, nuclear factor erythroid 2-related factor 2; *HO-1*, heme oxygenase; *GRP78*, Glucose-regulated protein 78; *ATF6*, activating transcription factor 6; *PERK*, PKR-like endoplasmic reticulum kinase; *IRE1*, inositol-requiring enzyme 1; *CHOP*, C/EBP homologous protein; *BCL-2*, B-cell lymphoma 2.

Redox and ER stress-associated gene expression was evaluated by RT-PCR. PA administration induced *GRP78*, *PERK*, *IRE1*, and *ATF6* expression in Huh-7 cells. 5-ALA (200 μM) induced *HO-1* expression, but not by inducing the upstream nuclear factor erythroid 2–related factor 2 (*NRF2*), the master regulator of cellular redox homeostasis (Figure 2a,b). 5-ALA significantly induced *HO-1* expression and significantly decreased *GRP78*, the key sensing molecule of ER stress (Figure 2c). The expression of *ATF6*, one of the three stress transducers, also decreased with 5-ALA (Figure 2d). *PERK* and *IRE1* were not altered by 5-ALA (Figure 2e,f). 5-ALA did not alter the expression level of the pro-apoptotic C/EBP homologous protein (*CHOP*) (Figure 2g). Instead, anti-apoptotic B-cell lymphoma 2 (*BCL-2*) was significantly increased (Figure 2h).

### 2.3. 5-ALA Decreased Caspase 3/7 Activity and Lipoapoptosis

**Figure 3:**
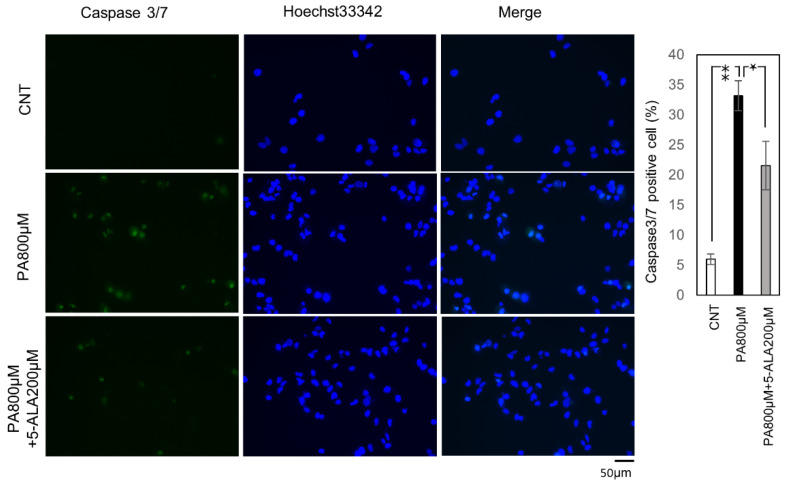
Fluorescein active Caspase-3/7 staining. Huh-7 cells treated with 1% BSA were the controls, and palmitic acid 800 μM with or without 5-ALA 200μM the treatment groups. The results were expressed as the ratio compared with the control. The quantification is based on ten randomly captured fields in each group. Bars indicate average ± SEM. * *p* < 0.05, ** *p* < 0.01. CNT, control; PA, palmitic acid.

**Figure 4:**
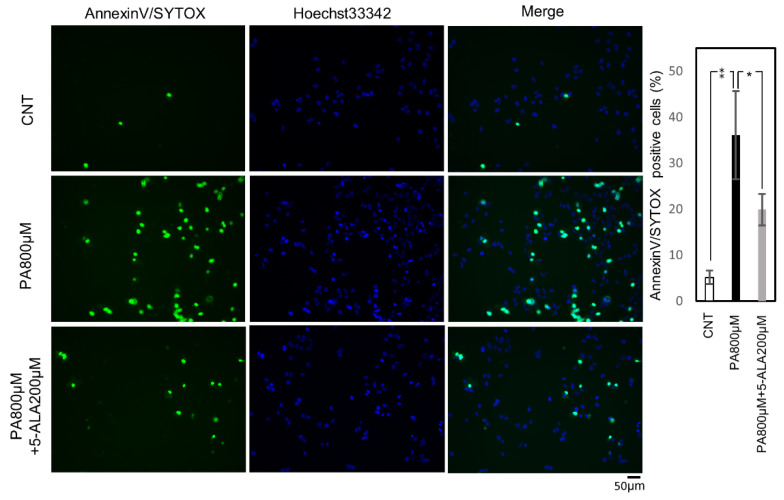
Cell death assay. Huh-7 cells were treated with 1% BSA for the control, and palmitic acid 800 μM with or without 5-ALA 200 μM for the treatment groups. The results were expressed as the ratio compared with the control. The quantification is based on ten randomly captured fields in each group. Bars indicate average ± SEM. * *p* < 0.05, ** *p* < 0.01. CNT, control; PA, palmitic acid.

Then we evaluated the effect of 5-ALA on lipoapoptotis induced by steatosis. First, the activity of Caspase-3/7, a cysteine-aspartic acid protease that can directly execute apoptosis, was evaluated. The cells with active Caspase 3/7 were significantly increased by PA (800 μM) administration, and 5-ALA (200 μM) significantly decreased this activity (Figure 3). Subsequently, we evaluated cell death using Annexin V/SYTOX staining. Annexin/SYTOX-positive cells were significantly increased by PA (800 μM) administration, and co-administration of PA and 5-ALA (200 μM) significantly decreased the positive cells (Figure 4). 

## 3. Discussion

In this study, we demonstrated that 5-ALA could attenuate GRP78 expression and suppress lipoapoptosis via HO-1 induction. ER stress-mediated cellular apoptosis is an essential mechanism of NASH pathogenesis and progression [28]. However, therapeutic approaches directly impacting ER stress remain limited [29]. 

Our study showed that PA increased the expression of the *GRP78*, *ATF6*, *PERK*, and *IRE1*, and consequently induced apoptosis by activating Caspase3/7 in Huh-7 cells. This result means PA-induced ER stress-activated UPR, which resulted in lipoapoptosis. 

**Figure 5:**
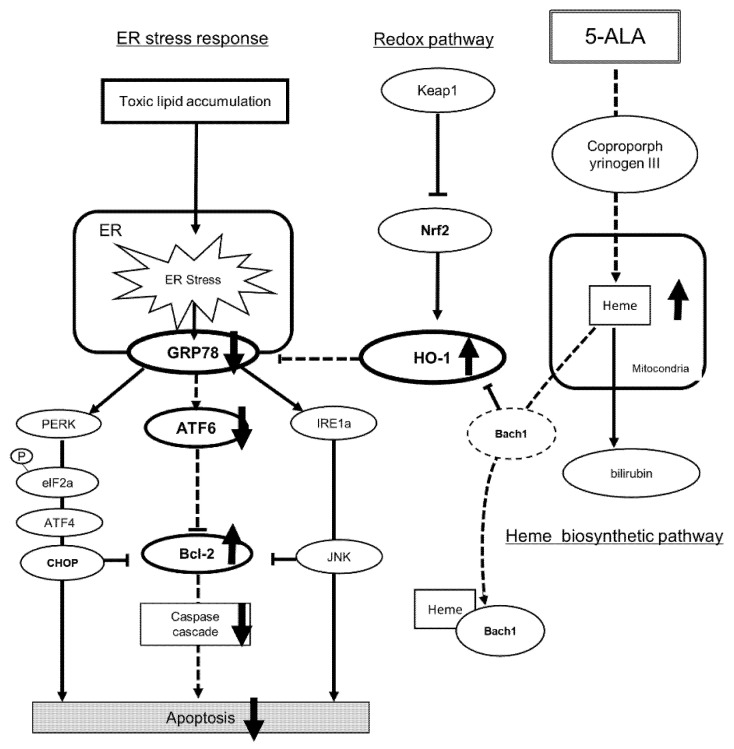
Scheme of how 5-ALA regulates ER stress and attenuates apoptosis. 5-ALA induces HO-1 expression when synthesized to heme. Bach1 is inactivated by direct binding to a higher concentration of heme than Bach1, which allows for increased expression of HO-1. Increased HO-1 downregulates GRP78 expression. Decreased GRP78 expression attenuates ATF6, subsequently increasing Bcl-2 expression and decreasing caspase activity, which attenuates apoptosis. 5-ALA, 5-aminolevulinic acid; CNT, control group; PA, palmitic acid group; Bach1, BTB and CNC homology; NRF2, nuclear factor erythroid 2-related factor 2; HO-1, hem oxygenase-1; GRP78, Glucose-regulated protein 78; PERK, plasmic reticulum kinase; IRE1, inositol-requiring enzyme 1; ATF6, activating transcription factor 6; Bcl-2, B-cell lymphoma 2.

Our results also showed that 5-ALA increased *HO-1* expression and reduced upregulated *GRP78* expression. Unexpectedly, our results also indicate that 5ALA upregulated *HO-1*, but not by inducing *NRF2*. HO-1 is a key molecule in the Nrf-2/HO-1 redox system and is regulated by Nrf-2 [30]. However, under conditions with a higher concentration of heme, HO-1 is inactivated by direct binding to BTB and C NC homology 1 (Bach1), which allows for increased HO-1 expression [19,31]. This pathway explains how 5-ALA can directly regulate HO-1 expression (Figure 5). 

Several studies have demonstrated that HO-1 induction could suppress steatohepatitis in vitro and in vivo by inducing an antioxidant pathway, suppressing cytokine production, and modifying fatty acid turnover [32,33]. The primary mechanism of HO-1 suppressing steatohepatitis is by attenuating oxidative stress.

GRP78, an ER-located heat shock protein 70 molecular chaperone, plays a pivotal role in activating the UPR to restore ER homeostasis [34]. Regulating GRP78 is crucial to regulating ER stress. However, only BiP inducer X (BIX) reduces ER stress by directly inducing GRP78 [35]. In stressed cells, once GRP78 is occupied by unfolded and misfolded proteins, the UPR transducers (PERK, IRE1, and ATF6) are released from GRP78 and activated [6]. If the ER stress persists, UPR switches from pro-survival to pro-apoptosis via CHOP in association with down-regulation of Bcl-2 [36]. Increased free fatty acids, particularly saturated fatty acids, have been linked to UPR activation [13]. PA is the most prevalent circulating saturated fatty acid, and it reportedly induces apoptosis due to ER stress by increasing CHOP and activating Caspase 3 expression [37,38]. Sustained lipid accumulation exceeds the processing capabilities of ER, which leads to mitochondrial dysfunction and triggers lipoapoptosis. Our results did not demonstrate any statistical difference in CHOP and Bcl-2 transcription by PA administration. However, we captured apoptosis by demonstrating Caspase 3/7 activation and increased cell death, a phenomenon also found in the NASH animal model [39]. 

In the present study, only *ATF6* was decreased by 5-ALA. High expression of active ATF6 reportedly down-regulated Bcl-2 mRNA expression in ER stress [40]. It implies an interaction between ATF6 and Bcl-2 with each other in UPR. The anti-apoptotic Bcl-2 family plays a central role in the regulation of UPR [41]. Upregulation of Bcl-2 means protection for stressed cells from cell death [42]. Our results demonstrated that *BCL-2* is upregulated in Huh-7 cells, and it may be induced by reducing *ATF6* expression by 5-ALA.

Islam et al. [43] have recently demonstrated in heat-stressed bovine mammary epithelial cells (MECs) that 5-ALA pretreatment significantly suppressed GRP78. The same group recently reported that 5-ALA increased BCL-2 and reduced cleaved caspase-3 protein expression using PA-treated bovine MECs [44]. These findings are consistent with our results. 

Sharmin et al. [44] concluded that upregulated HO-1 directly suppressed phosphorylated PERK; however, GRP78 is upstream of PERK and regulates PERK activation [35]. The mechanism of 5-ALA in regulating GRP78 is still not fully explained. Therefore, we speculated that highly upregulated HO-1 would directly affect GRP78 regulation. Some reports demonstrated that GRP78 overexpression increased Nrf2 and HO-1 expression [45,46,47]. HO-1 induction by losartan inhibits GRP78 expression [48]. These findings indicate that there are direct interactions between the GRP78 and Nrf2/HO-1 system. Our results demonstrate that *HO-1* upregulated by 5-ALA is not only protective against oxidative stress but also effective against ER stress via attenuating GRP78 expression. 

The limitations of our study are that it only evaluated selected gene expressions and did not include immune blots and animal experiments. However, the selected genes are the key genes of the redox pathway and UPR. Therefore, we could demonstrate the therapeutic potential of 5-ALA on hepatic steatosis from the regulation of GRP78. The induction of HO-1 is a promising therapeutic target for both redox pathways and the ER stress response. Additional investigation using animal models is warranted.

## 4. Materials and Methods

### 4.1. Reagents 

Palmitic acid (PA, P5585-10G; Sigma-Aldrich, St. Louis, MO, USA) was obtained from Merck KGaA. 5-ALA (AL-05-1, Cosmo Bio Co., Tokyo, Japan) from Cosmo Bio Co., Ltd. 

### 4.2. Cell Lines and Cultures

We selected Huh-7 (well-differentiated human hepatocellular carcinoma cell line) among the human in vitro models of hepatocyte [49]. We obtained Huh-7 from the JCRB Cell Bank. The Huh-7 cell line was grown without antibiotics in the Dulbecco’s Modified Eagle’s Medium (DMEM; FUJIFILM Wako Pure Chemical Corporation, Osaka, Japan) supplemented with 10% fetal bovine serum (FBS; Biosera, Kansas City, MO, USA) and 1% L-glutamine solution (FUJIFILM Wako Pure Chemical Corporation, Osaka, Japan). The cells were cultured in a humidified incubator with 5% CO_2_ at 37 °C. PA was dissolved in isopropanol at 37 °C to a concentration of 100 mM according to other studies [50,51]. The dissolved PA solution was mixed with DMEM containing 1% fetal bovine serum albumin (BSA; FUJIFILM Wako Pure Chemical Corporation, Osaka, Japan), and the final concentration at the time of administration was adjusted to 200, 400, 600, and 800 μM in DMEM containing 10% fetal bovine serum. 5-ALA was dissolved in serum-free DMEM to a concentration of 1 mM, and the final concentration at the time of administration was adjusted to 200 μM in DMEM containing 10% fetal bovine serum. The final concentration of BSA was adjusted to 200 μM in D-MEM containing 10% fetal bovine serum. 1% BSA was administered to the normal controls in each group in equal volume with the palmitic acid solution.

### 4.3. RNA Extraction and Real-Time PCR

Huh-7 cells were seeded into a 6-well plate at a density of 3 × 10^5^ cells/mL (1 mL/well). Following incubation for 24 h, the cells were treated with 1% BSA and 800 μM of PA with or without 200 μM of 5-ALA for eight hours. The total RNA from cultured cells was extracted using the miRNeasy Mini Kit (217004; Qiagen, Hilden, Germany). The RNA was quantified using the Biospec-nano spectrophotometer (Shimadzu, Kyoto, Japan). The extracted RNA samples were stored at −80 °C. cDNAs were prepared from total RNA using the High-Capacity cDNA Reverse Transcription Kit (4374966; Thermo Fisher Scientific, Waltham, MA, USA). The reverse transcription (RT) reactions were performed in aliquots containing 2 µg of total RNA, 1 X RT buffer, 4 mM dNTP mix, 1 X RT random primer, 50 units of MultiScribe reverse transcriptase, 20 units of RNase inhibitor, and made to a final volume of 20 μL with nuclease-free water. The reactions proceeded at 25 °C for 10 min, followed by 37 °C for 120 min, and 85 °C for 5 min. The RT-PCR reaction was performed in 20 μL aliquots containing 1 μL RT products with 4 μL LightCycler^®^ FastStart DNA Master PLUS SYBR Green I (03515869001; Roche Diagnostics, Basel, Switzerland), 0.5 μM of each primer, and 14.6 μL nuclease-free water. Analyses were run on the Real-Time PCR Light Cycler^®^ 1.5 Complete System (Roche Diagnostics, Basel, Switzerland). Thermal cycling was initiated with the first denaturation step at 95 °C for 10 min, followed by 45 cycles of 95 °C for 10 s, 60 °C for 10 s, and 72 °C for 10 s. The cycle threshold (Ct) was recorded for mRNA by LightCycler^®^ Software version 3.5.28 (Roche Diagnostics, Basel, Switzerland), and β-actin was used as the endogenous control for data normalization. The relative expression was calculated using the formula 2^−ΔΔCt^ =2^−(ΔCt, reagent treatment − ΔCt, control)^. The primers used were shown in Table 1.

### 4.4. Lipid Staining

Huh-7 cells were seeded into a 96-well plate at a density of 1 × 10^4^ cells/mL (200 µL/well). Following incubation for 24 h, the cells were treated with 1% BSA or different concentrations of PA for eight hours. Lipi-Green (LD-02, Dojindo Laboratories, Kumamoto, Japan), a lipid droplet-specific fluorescent probe for live-cell imaging, was used for lipid staining according to the manufacturer’s protocol. The plates were analyzed with an All-in-One Fluorescence Microscope BZ-X800 (KEYENCE, Osaka, Japan). Pixel intensity was measured using ImageJ software version 1.52a (Wayne Rasband; National Institutes of Health, Bethesda, MD, USA) on 20 random cells (magnification, ×20) according to the manufacturer’s instructions.

### 4.5. Quantification of Caspase 3/7 Activity

Huh-7 cells were seeded into a 96-well plate at a density of 1 × 10^4^ cells/mL (200 µL/well), followed by incubation for 24 h and treatment with 1% BSA and 800 μM of PA with or without 200 μM of 5-ALA for four hours. Caspase activity was evaluated by staining with Cell Even Caspase-3/7 Green Detection Reagent (C10423, Thermo Fisher Scientific, Waltham, MA, USA) according to the manufacturer’s protocol. The slides were analyzed with an All-in-One Fluorescence Microscope BZ-X800 (KEYENCE, Osaka, Japan). Caspase 3/7 positive cells were quantified by counting Caspase-3/7 Green positive nuclei and Hoechst 33342 positive nuclei in ten random microscopic fields (20×). The ratio was then calculated.

### 4.6. Quantification of Cell Death

Cell death was evaluated by staining with the Annexin V-FITC Apoptosis Detection Kit Plus (BioVision, Milpitas, CA, USA) and the Hoechst 33342 stain (Thermo Fisher Scientific, Waltham, MA, USA). Huh-7 cells were seeded into a 96-well plate at a density of 1 × 10^4^ cells/mL (200 µL/well), followed by incubation for 24 h and treatment with 1% BSA and 800 μM of PA with or without 200 μM of 5-ALA for four hours. Annexin-V with SYTOX green staining was performed per the manufacturer’s protocol. The plates were analyzed with an All-in-One Fluorescence Microscope BZ-X800 (KEYENCE, Osaka, Japan). Dead cells were quantified by counting Annexin-V and SYTOX Green positive nuclei and Hoechst 33342 positive nuclei in ten random microscopic fields (20×) and the ratio was then calculated.

### 4.7. Statistics

All data represent three or more independent experiments using cells from a minimum of three separate isolations. Skewness-Kurtosis was used to verify the data distribution. Welch’s *t*-test was used for comparison between two groups. One-way ANOVA was used for comparison between groups. Tukey’s test was applied for multiple comparisons. Statistical analyses were performed using StatFlex software (Windows ver. 6.0; Artech LLC, Osaka, Japan). *p* < 0.05 was considered a statistically significant difference, and all data are expressed as the mean ± SEM.

## 5. Conclusions

5-ALA has therapeutic potential for hepatic steatosis by suppressing ER stress and lipoapoptosis by attenuating GRP78 by HO-1 induction. It has the potential to be a candidate for drug repositioning; however, further investigation is warranted.

## Figures and Tables

**Table 1:** Sequences of Primers.

Gene	Fwd (5′ to 3′)	Rev (5′ to 3′)
*NRF2*	CAGCGACGGAAAGAGTATGA	TGGGCAACCTGGGAGTAG
*HO-1*	GCCAGCAACAAAGTGCAAG	GAGTGTAAGGACCCATCGGA
*GRP78*	AAGGGGAACGTCTGATTGGC	TGGATGAGGAAAACCGGTCG
*ATF6*	TCCTCGGTCAGTGGACTCTTA	CTTGGGCTGAATTGAAGGTTTTG
*PERK*	GGAAACGAGAGCCGGATTTATT	ACTATGTCCATTATGGCAGCTTC
*IRE1*	GCCGAAGTTCAGATGGAATC	ATCTGCAAAGGCCGATGA
*CHOP*	CCTCTGCCGTATCACCACAG	GGGCTGTGCTGCTCTTTAGA
*BCL-2*	CTGGTGGGAGCTTGCATCAC	ACAGCCTGCAGCTTTGTTTC
*β-Actin*	GCATCCTCACCCTGAAGTA	TGTGGTGCCAGATTTTCTCC

NRF2, nuclear factor erythroid 2-related factor 2; HO-1, hem oxygenase-1; GRP78, glucose-regulated protein 78; ATF6, activating transcription factor 6; PERK, plasmic reticulum kinase; IRE1, inositol-requiring enzyme 1; CHOP, C/EBP homologous protein; BCL-2, B-cell lymphoma 2.
